# Chaperone activity of niflumic acid on ClC-1 chloride channel mutants causing myotonia congenita

**DOI:** 10.3389/fphar.2022.958196

**Published:** 2022-08-11

**Authors:** Concetta Altamura, Elena Conte, Carmen Campanale, Paola Laghetti, Ilaria Saltarella, Giulia Maria Camerino, Paola Imbrici, Jean-François Desaphy

**Affiliations:** ^1^ Dept. of Biomedical Sciences and Human Oncology, School of Medicine, University of Bari Aldo Moro, Bari, Italy; ^2^ Dept. of Pharmacy-Drug Sciences, University of Bari Aldo Moro, Bari, Italy

**Keywords:** myotonia congenita, ClC-1 chloride channel, niflumic acid, drug repurposing, precision medicine

## Abstract

Myotonia congenita (MC) is an inherited rare disease characterized by impaired muscle relaxation after contraction, resulting in muscle stiffness. It is caused by loss-of-function mutations in the skeletal muscle chloride channel ClC-1, important for the stabilization of resting membrane potential and for the repolarization phase of action potentials. Thanks to *in vitro* functional studies, the molecular mechanisms by which ClC-1 mutations alter chloride ion influx into the cell have been in part clarified, classifying them in “gating-defective” or “expression-defective” mutations. To date, the treatment of MC is only palliative because no direct ClC-1 activator is available. An ideal drug should be one which is able to correct biophysical defects of ClC-1 in the case of gating-defective mutations or a drug capable to recover ClC-1 protein expression on the plasma membrane for trafficking-defective ones. In this study, we tested the ability of niflumic acid (NFA), a commercial nonsteroidal anti-inflammatory drug, to act as a pharmacological chaperone on trafficking-defective MC mutants (A531V, V947E). Wild-type (WT) or MC mutant ClC-1 channels were expressed in HEK293 cells and whole-cell chloride currents were recorded with the patch-clamp technique before and after NFA incubation. Membrane biotinylation assays and western blot were performed to support electrophysiological results. A531V and V947E mutations caused a decrease in chloride current density due to a reduction of ClC-1 total protein level and channel expression on the plasma membrane. The treatment of A531V and V947E-transfected cells with 50 µM NFA restored chloride currents, reaching levels similar to those of WT. Furthermore, no significant difference was observed in voltage dependence, suggesting that NFA increased protein membrane expression without altering the function of ClC-1. Indeed, biochemical experiments confirmed that V947E total protein expression and its plasma membrane distribution were recovered after NFA incubation, reaching protein levels similar to WT. Thus, the use of NFA as a pharmacological chaperone in trafficking defective ClC-1 channel mutations could represent a good strategy in the treatment of MC. Because of the favorable safety profile of this drug, our study may easily open the way for confirmatory human pilot studies aimed at verifying the antimyotonic activity of NFA in selected patients carrying specific ClC-1 channel mutations.

## 1 Introduction

A delay in muscle relaxation after a prolonged contraction is the main characteristic of myotonia congenita (MC), one of the most common skeletal muscle channelopathies affecting patients with a prevalence of 1–9/100.000 worldwide (Dunø and Vissing, 2005). Clinically, MC should be suspected in individuals who presented episodes of muscle stiffness, myotonic contraction elicited by muscle percussion, and stiffness amelioration by brief exercise (warm-up phenomenon) (Stunnenberg et al., 2020). MC is caused by loss of function mutations in the *CLCN1* gene, coding for the voltage-gated chloride channel ClC-1. This channel is expressed almost exclusively in the skeletal muscle, where it represents the main contributor to the large resting membrane conductance that is crucial for the correct membrane repolarization and propagation of action potential (Maggi et al., 2021).

To date, more than 300 pathogenic mutations are identified in the *CLCN1* gene (Human Gene Mutation Database, 2022; http://www.hgmd.cf.ac.uk/ac/index.php), which are scattered over the entire human ClC-1 protein, including the cytosolic N- and C-terminal regions and the transmembrane domains. Most of the mutations functionally characterized so far are predicted to alter ClC-1 channel gating properties or to reduce channel surface expression, classifying them in “gating-defective” or “expression-defective” MC mutations. The former includes various alterations of channel functions, including a shift of the voltage dependence of fast or slow gating, reduced single-channel conductance, and altered ion selectivity. The latter can be a consequence of altered synthesis, defective folding, impaired membrane trafficking, or defective stability at the plasma membrane (Weinberger et al., 2012; Desaphy et al., 2013; Imbrici et al., 2016; Jeng et al., 2020). In both cases, the reduced chloride influx hampers membrane repolarization after an action potential and generates the typical myotonic runs of action potentials (Altamura et al., 2020a).

Given the pathogenesis of MC, the ideal drug to treat this disorder should be able to restore the sarcolemma chloride current. Unfortunately, no drug is known with such a mechanism of action. Hence, the current therapeutic approach for myotonia relies on drugs able to dampen the skeletal muscle hyperexcitability through inhibition of voltage-gated sodium channels, thus relieving patients’ symptoms. For instance, the effectiveness of mexiletine and lamotrigine has been demonstrated in randomized clinical trials, while other sodium channel blockers have proved beneficial in open cohort studies and case reports (Statland et al., 2012; Lo Monaco et al., 2015; Andersen et al., 2017; Arnold et al., 2017; Stunnenberg et al., 2018; Desaphy et al., 2021). These drugs can be effective in reducing muscle stiffness and transient weakness in MC patients. Mexiletine is the only drug indicated for MC, but its use is limited by country-dependent availability, contraindications, intolerable side effects, and suboptimal response in more than 30 % of patients. In this context, we are searching for drugs that may correct the defect of myotonic ClC-1 mutants, focusing here our attention on potential pharmacological chaperones that may increase the plasma membrane expression of trafficking-defective mutants.

To date, at least 11 MC mutations are associated with ClC-1 proteostasis impairment (Altamura et al., 2020b; Jeng et al., 2020). This number is likely underestimated as targeted biochemical analyses are lacking for a number of other ClC-1 mutants (Jeng et al., 2020). In our experience, 10 out of 24 ClC-1 functionally characterized mutations revealed a drastic reduction of chloride current density, suggesting a defect in ClC-1 proteostasis (Desaphy et al., 2013; Imbrici et al., 2015; Altamura et al., 2018a; Altamura et al., 2020b). In the current study, we selected the well-known *p*. A531V and the recently characterized *p*. V947E mutations for pharmacological investigation. The *p*. A531V mutation located in helix O does not affect ClC-1 gating but causes enhanced proteasomal protein degradation and reduced membrane expression (Papponen et al., 2008; Desaphy et al., 2013; Lee et al., 2013; Chen et al., 2015). Similarly, the *p*. V947E mutation in the C-terminus showed a 55% reduction of chloride currents but no change in voltage dependence compared to WT (Altamura et al., 2018a).

Because activators of ClC-1 channels are dramatically lacking, we tested the chaperone activity of a potent but reversible inhibitor of the channel, the nonsteroidal anti-inflammatory drug niflumic acid (NFA), on A531V and V947E mutant channels. Although the inhibitory activity is neither required nor desirable, many pharmacological chaperones are reversible competitive inhibitors or antagonists of their target proteins. In this study, we show that NFA reversibly blocked the ClC-1 channel when the drug was applied acutely, whereas it can act as a pharmacological chaperone after prolonged exposure.

The results provide evidence that small ClC-1 channel ligands might be useful for restoring the activity of poorly expressed MC mutants, with the perspective to offer a personalized medicine to the myotonic individuals carrying such mutations.

## 2 Materials and methods

### 2.1 Site-directed mutagenesis, cell culture, and transient transfection

The c.1592C>T (*p*.A531V) and c.2840T>A (*p*.V947E) mutations were, respectively, introduced into the plasmid pRcCMV-hClC-1 and pcDNA3.1/V5-His TOPO TA containing the full-length wild-type (WT) hClC-1 cDNA using the QuickChange™ site-directed mutagenesis kit (Stratagene Cloning Systems), as previously described (Desaphy et al., 2013; Altamura et al., 2018a). Sequencing of the complete coding region of the cDNA was performed to exclude polymerase errors. Human embryonic kidney (HEK293) cells were grown in Dulbecco’s modified Eagle’s medium Plus (DMEM+) containing DMEM, 10% fetal bovine serum, 5% penicillin–streptomycin, and 5% L-Glutamine and were maintained at 37°C in a 5% CO_2_/95 % O_2_ atmosphere. Transient transfection was performed by using the calcium-phosphate precipitation method. A mixture of hClC-1 (0.5 μg/ml) and CD8 reporter plasmids (0.1 μg/ml) was added to HEK293 cells plated into 100 mm dishes. After 24 h, HEK cells were seeded into 60 mm dishes for patch-clamp experiments or maintained in 100 mm dishes for biochemical experiments. For investigating chaperone effects, cells were then incubated for 24 h in DMEM + supplemented with 30–50–100 μM NFA (Sigma-Aldrich) dissolved in dimethylsulfoxide (final DMSO concentration 0.03%) or with 0.03% DMSO alone. For investigating acute effects, the HEK cells were seeded into 60 mm dishes containing DMEM+ and were used for patch-clamp experiments 48–72 h after transfection.

### 2.2 Patch-clamp experiments

Only transfected HEK293 cells decorated with anti-CD8 antibody-coated microbeads (Dynabeads M450, Invitrogen) were used for standard whole-cell patch-clamp recordings at room temperature (∼20°C) using an Axopatch 200B amplifier (Axon Instruments). The extracellular solution contained (in mM) 140 NaCl, 4 KCl, 2 CaCl_2_, 1 MgCl_2_, and 5 HEPES, and the pH was adjusted to 7.4 with NaOH. The pipette solution contained (in mM) 130 CsCl, 2 MgCl_2_, 5 EGTA, and 10 HEPES, and the pH was set at 7.4 with CsOH. The equilibrium potential for chloride ions was about −2.8 mV, and cells were clamped at the holding potential (HP) of 0 mV. Patch-clamp pipettes were pulled from borosilicate glass using the PC-100 puller (Narishige) and had a resistance lower than 3 MΩ. Currents were low-pass-filtered at 2 kHz and digitized with sampling rates of 50 kHz using a Digidata 1550B AD/DA converter (Axon Instruments). Chloride currents were recorded ∼2 min after achieving the whole-cell configuration to allow the pipette solution to equilibrate with the intracellular solution. The voltage-dependent channel activity was measured by applying specific voltage step pulses from −150 mV to + 150 mV in 10 mV intervals. Each voltage step was followed by a voltage step at −105 mV to evoke tail currents. Voltage steps were applied every 3 s to allow complete recovery of the current amplitude at the HP between two pulses.

The instantaneous and steady-state current–voltage relationships were drawn by measuring instantaneous and steady-state current densities (pA pF–1) at the beginning (∼1 ms) and end (∼390 ms) of each voltage step. The total apparent open probability (Po) for WT and mutant channels was obtained from the normalized peak tail currents. The voltage dependence of channel activation was determined by plotting Po as a function of the voltage of the conditioning test pulses. The data points were fitted with a Boltzmann function:

$$\mathrm{Po}\left(V\right)=\mathrm{Pmin}+\left(\mathrm{1-Pmin}\right)/\left\{\mathrm{1+exp}\left[\left({\mathrm{V-V}}_{\mathrm{0.5}}\right)\right]/K\right\},$$

where Pmin is the minimal value of Po, V_0.5_ is the half-maximal activation potential, and k is the slope factor. Data were analyzed offline by using pClamp 11.1 (Axon Instruments) and SigmaPlot 10 (Systat Software GmbH) software.

To study the acute effects of NFA (50 µM), the cells were perfused with a control or drug-supplemented bath solution during patch-clamp recording. To study the effects after 24 h of incubation, cells were washed several times with a control bath solution before recording whole-cell chloride currents.

### 2.3 ClC-1 protein expression

The total ClC-1 protein expression was measured in transfected HEK293 cells after 24 h incubation with 50 μM NFA or 0.03 % DMSO. After incubation, cells were harvested in 200 μl of cold RIPA buffer (20 mM Tris-HCl, 150 mM NaCl, 1,5% Non-idet P-40, 100 mM sodium orthovanadate, 10 mg/ml PMSF, and a protease inhibitor cocktail) and placed for 10 min in ice. To complete cell lysis, suspensions were passed through the needle of a syringe 10 times. After 15 min in ice, cell lysates were centrifuged at 14,000 rpm for 30 min at 4°C, and the supernatant was collected. Total protein amounts were quantified by using a BCA protein assay kit (Bio-Rad, Hercules, CA, United States).

### 2.4 Biotinylation of cell-surface proteins

Biotinylation experiments were performed to measure plasma and cytosolic ClC-1 protein expression in WT and V974E transfected cells after 24 h incubation with 50 μM NFA or 0.03% DMSO using a Cell Surface Protein Isolation Kit (Pierce, Rockford, IL, United States). Briefly, cells were washed twice with ice-cold PBS, and sulfosuccinimidyl-2-(biotinamido) ethyl-1,3-dithiopropionate (Sulfo-NHS-SS-biotin) was added to label cell-surface proteins. A quenching solution was used to quench excess biotin. Cells were treated with the lysis buffer and centrifuged at 10,000 g for 2 min at 4°C. The clear supernatant was reacted with immobilized NeutrAvidin gel slurry, in columns, for 60 min at room temperature. After centrifugation, cytoplasmic proteins were recovered from the flow-through, while surface proteins were eluted in a sample buffer containing dithiothreitol. Protein amounts were quantified by using a PierceTM BCA Protein Assay Kit, Reducing Agent Compatible (Thermo Scientific, United States), and then used for western blot experiments.

### 2.5 Western blot analysis

Total proteins or surface and cytoplasmic proteins (8 μg) were separated on a 10% sodium dodecyl sulfate polyacrylamide gel electrophoresis and transferred onto nitrocellulose membranes for 1 h at 200 mA (SemiDry transferblot; Bio-Rad). Afterward, membranes were blocked for 2 h with 0.2 M Tris-HCl, 1.5 M NaCl, pH 7.4 buffer (TBS) that contained 5% nonfat dry milk and 0.5% Tween-20 and incubated overnight at 4°C with rabbit anti-CLCN1 antibody (ab189857, Abcam) diluted 1:500 and monoclonal mouse anti-Actin (Sc-47778, Santa Cruz Biotechnology) diluted 1:300 with TBS containing 5% nonfat dry milk. Then, membranes were washed with TBS containing 0.5% Tween-20 (TTBS) and incubated for 1 h with goat anti-rabbit and anti-mouse IgG conjugated to horseradish peroxidase (Biorad). After a wash with TTBS, membranes were developed with a chemiluminescent substrate (Clarity Western ECL Substrate; Bio-Rad) and visualized on a Chemidoc imaging system (Bio-Rad).

Densitometric scans of western blots were quantified by using ImageJ Lab software (Bio-Rad), which allows the chemiluminescence detection of each experimental protein band to obtain the absolute signal intensity automatically adjusted by subtracting the local background. For total protein expression, density was standardized as the ratio of the ClC-1 signal to the cognate β-actin signal. For the biotinylation assay, the distribution of ClC-1 was quantified by calculating the ratio of surface proteins to the sum of surface and cytoplasmic proteins. Quantitative analysis was performed from three independent experiments.

### 2.6 Statistical analysis

Data are shown as box-and-whisker plots with the central line in the box representing the median and the dashed line representing the mean, while the box shows the interquartile range, with the whiskers indicating the 90th and 10th percentiles. The significance of the difference between the two groups was tested using Student’s t-test, whereas means from multiple groups were compared using the one-way ANOVA analysis, followed by Bonferroni’s t-test. All statistical analyses were performed using GraphPad Prism5 (GraphPad Software Inc.).

## 3 Results

### 3.1 Basic chloride current differences between wild-type, A531V, and V947E mutants

Previous studies have demonstrated that A531V and V947E mutant channels show a drastic reduction of chloride currents, although the voltage dependence appeared similar to that of WT (Desaphy et al., 2013; Lee et al., 2013; Altamura et al., 2018a). Such effects were confirmed in the current study, demonstrating that the instantaneous and steady-state current amplitudes were greatly reduced through the entire voltage range for A531V and V947E compared with WT (Table 1). The voltage dependence of activation of A531V and V947E mutant channels was similar to that of WT, confirming previous results (Table 1).

**TABLE 1:** Biophysical parameters of hClC-1 WT and MC mutants.

hClC-1	I_ist_ (−90 mV) (pA/pF)	I_ist_ (+60 mV) (pA/pF)	I_ss_ (−90 mV) (pA/pF)	I_ss_ (+60 mV) (pA/pF)	V_0.5_ (mV)
WT (*n* = 8)	−158 ± 51	53 ± 23	−64 ± 31	52 ± 25	−62 ± 15
A531V (*n* = 7)	−40 ± 46*	11 ± 9**	−12,2 ± 10**	9 ± 8**	−50 ± 12
V947E (*n* = 7)	−75 ± 5*	29 ± 13*	−31 ± 14*	17 ± 8*	−66 ± 14

Data are mean ± SD of the number of cells indicated in brackets. Statistical analysis was performed using one-way ANOVA, followed by Bonferroni’s test (** *p* <0.01 and * *p* <0.05 compared with WT).

### 3.2 Acute effect of niflumic acid on ClC-1 A531V and V947E mutants

We previously determined the concentration–response relationship of NFA block on WT chloride currents, demonstrating that the acute application of NFA inhibited the WT ClC-1 steady-state current with an IC_50_ of ∼97 μM at −90 mV and that this inhibition was rapid in onset and completely reversed in less than 5 min upon washout (Altamura et al., 2018b).

**FIGURE 1:**
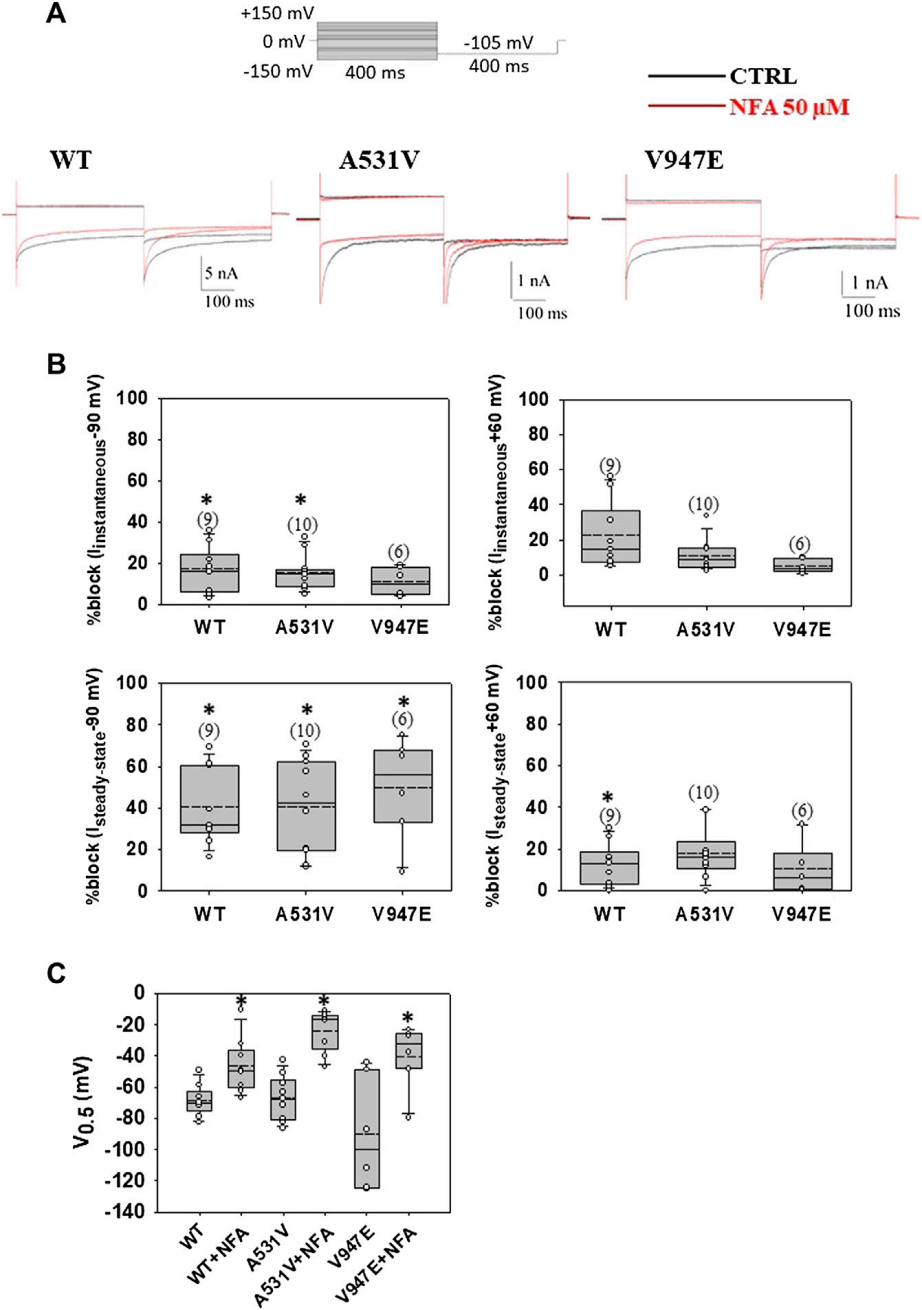
Acute application of NFA on WT, A531V, and V947E ClC-1 chloride channels. **(A)**. Representative traces of WT, A531V, and V947E chloride currents at −90 and +60 mV before (in black) and after (in red) the application of 50 µM NFA. **(B)**. Percentage of block of instantaneous and steady-state chloride current for WT, A531V, and V947E mutants measured at −90 mV and +60 mV induced by NFA 50 µM. **(C)**. Values of V_0.5_ (half-maximal activation potential) of WT, A531V, and V947E channels before and after the application of NFA. Each dot represents the V_0.5_ value obtained from a single recording. Data are shown as box-and-whisker plots. Median (solid line); mean (dash line); and 10th, 25th, 75th, and 90th percentiles are indicated. The number of examined cells is reported in brackets. Asterisk denotes a significant difference compared with the relative control condition (*p < 0.05).

Here, we evaluated the acute effect of NFA at the concentration of 50 μM on WT and mutant channels (Figure 1). As shown in Figure 1, the acute application of 50 μM NFA in HEK cells transfected with WT ClC-1 channels caused a significant reduction of steady-state chloride currents at −90 and +60 mV of 40 and 15 %, respectively (Figures 1A,B). The A531V and V947E mutants showed a sensitivity to 50 μM NFA similar to that of WT, suggesting that these mutations did not alter NFA binding to the ClC-1 chloride channel (Figures 1A,B). In addition, the acute application of NFA induced a significant shift of voltage dependence toward more positive potentials for WT (≈27 mV) and mutant channels (≈40 mV for A531V and ≈37 mV for V947E) (Figure 1C). Effects of NFA on WT and mutant channels were fully reversible (not shown).

### 3.3 Chaperone effect of niflumic acid on A531V and V947E ClC-1 mutants

**FIGURE 2:**
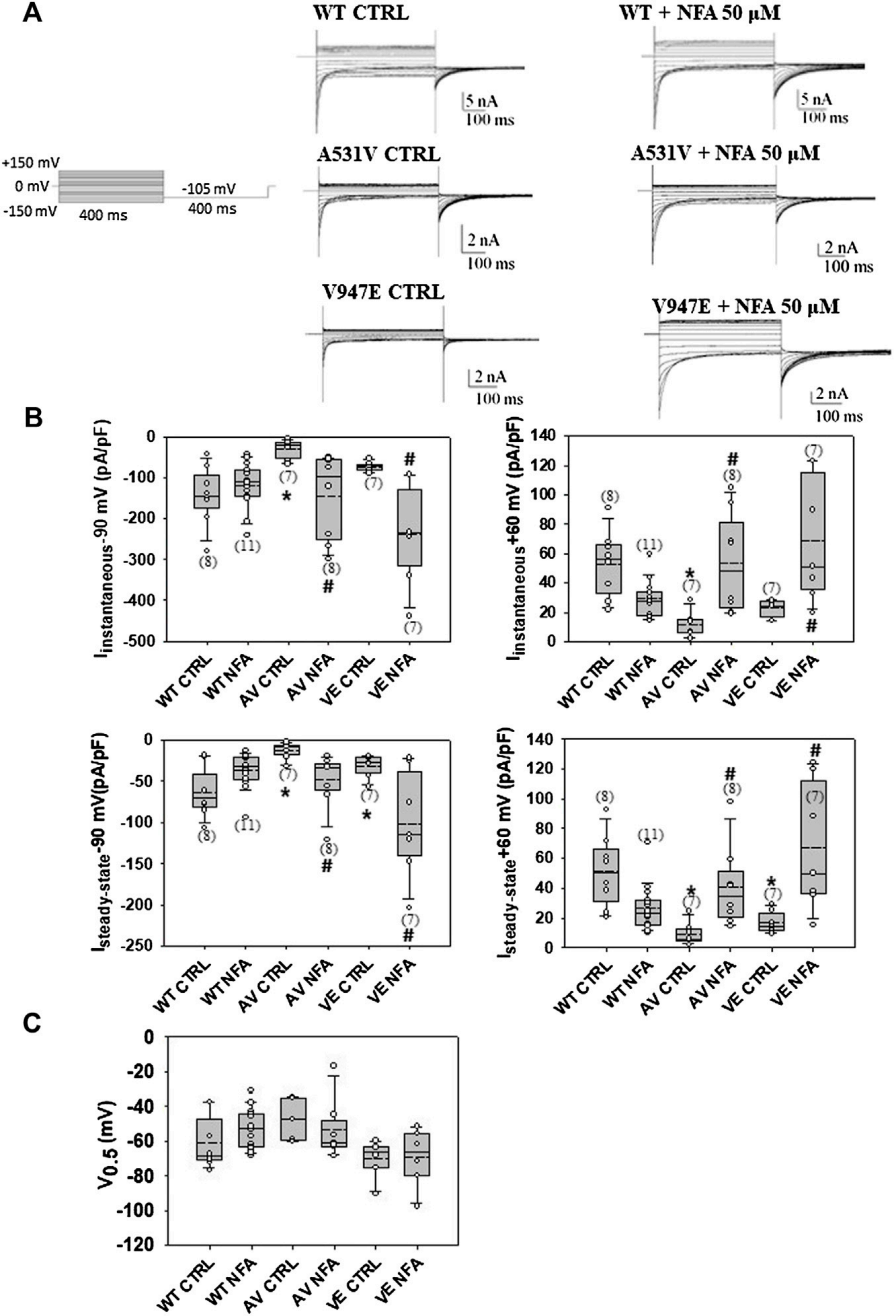
Chaperone effect of NFA on WT, A531V, and V947E ClC-1 chloride channels. **(A)**. Representative traces of WT, A531V, and V947E chloride currents before and after the incubation of 50 µM NFA for 24 h. **(B)**. Values of instantaneous and steady-state chloride currents of WT, A531V, and V947E mutants measured at −90 and +60 mV before and after the incubation with NFA 50 µM. **(C)**. Values of V_0.5_ (half-maximal activation potential) of WT, A531V, and V947E channels before and after the incubation of NFA. Each dot represents the V_0.5_ value obtained from a single recording. Data are shown as box-and-whisker plots. Median (solid line); mean (dash line); and 10th, 25th, 75th, and 90th percentiles are indicated. The number of examined cells is reported in brackets. (*at least *p* < 0.05 vs WT CTRL, # at least *p* < 0.05 vs its relative CTRL).

The chaperone effect of NFA was tested at the concentrations of 30, 50, and 100 μM because these values fall within the range of therapeutic blood concentrations used in humans (Schulz et al., 2012). Preliminary experiments performed on A531V mutant channels using 100 µM of NFA showed that NFA incubation induced an increase of A531V chloride currents more than 10-fold at −90 mV (Supplementary Figure S1). After 24 h of incubation with 50 μM NFA (and drug washout), the current density was not significantly altered for WT but showed a drastic significant increase of more than 5-fold for A531V and 3-fold for V974E, restoring the chloride current density similar to that of WT (Figures 2A,B). No significant difference was observed in the voltage dependence, suggesting that incubation with NFA increased protein membrane expression without altering the function of ClC-1 (Figure 2C). Conversely, the treatment of transfected cells with 30 µM NFA did not induce any significant effects on A531V and V947E chloride current densities (Supplementary Figure S2). The next biochemical experiments were thus performed using 50 µM NFA.

**FIGURE 3:**
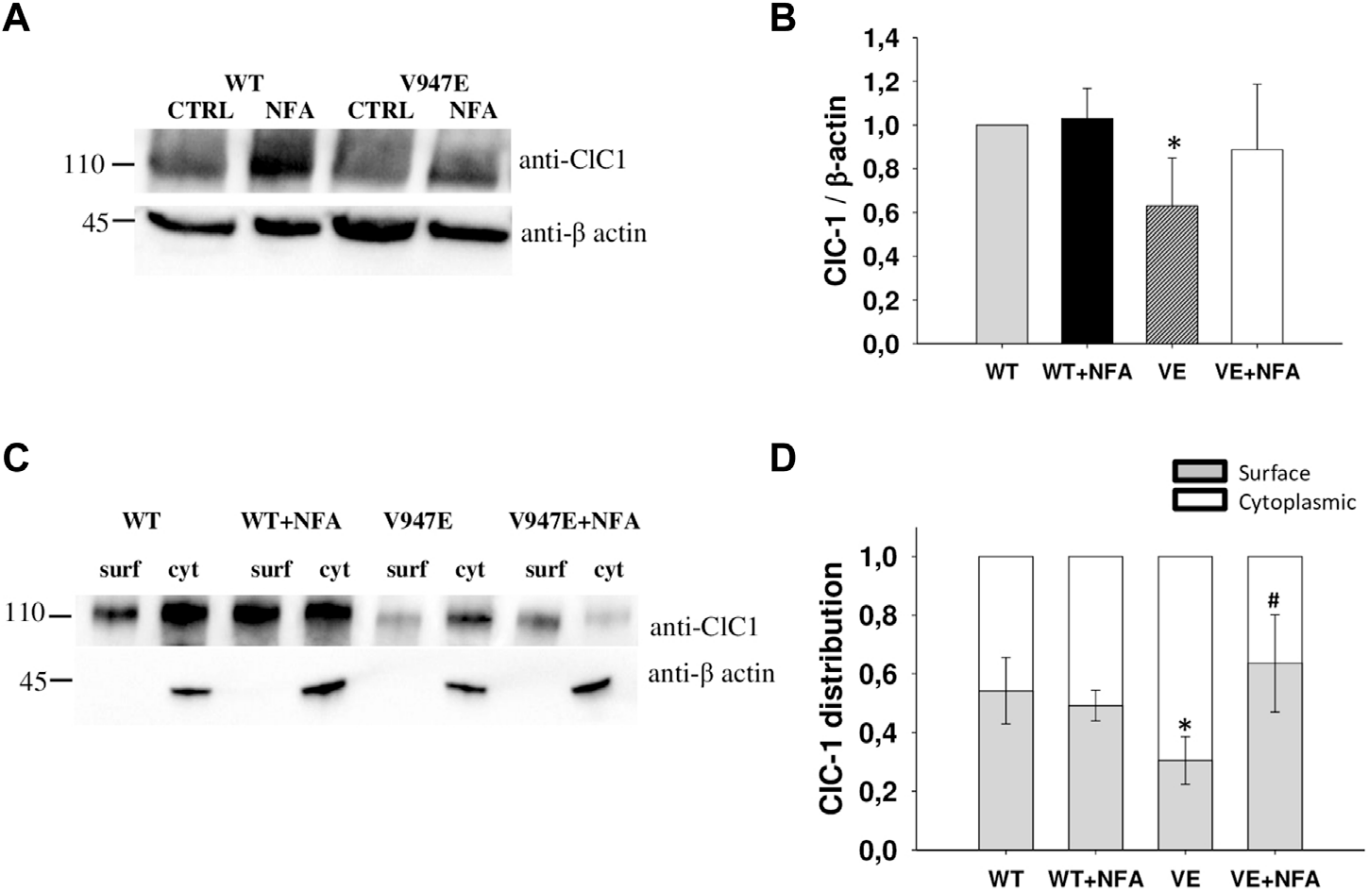
ClC-1 protein expression in HEK293T cells transfected with WT and V947E ClC-1 channels and effects of 50 µM NFA incubation for 24 h. **(A)**. Representative western blot of total ClC-1 and β-actin proteins from transfected HEK293T with WT and V947E in the absence or presence of 50 μM NFA. **(B)**. Quantification of ClC-1 total protein expression level. The ClC-1 signal of each column was standardized to the β-actin signal and normalized to WT on the same blot. Each bar is the mean ± S.E.M. from three independent experiments. Asterisks denote a significant difference (*p* < 0.05) between V947E in the control condition and after NFA treatment. **(C)**. Representative western blot of surface and cytoplasmic ClC-1 and β-actin proteins obtained from the biotinylation assay of transfected HEK293T cells incubated for 24 h in the absence or presence of 50 μM NFA. **(D)**. Quantification of surface and cytoplasmic ClC-1 protein distribution. Each bar is the mean ± SD from three independent experiments (*at least *p* < 0.05 vs WT in the control condition, # at least *p* < 0.05 vs V947E in the control condition).

Since a difference in current densities might be explained by different levels of channel protein expression, we studied the effects of NFA on WT and V947E mutant proteins using the western blotting analysis and the biotinylation assay. Quantitative analysis of the total protein amount showed a significant difference between WT and V947E mutant channels, with a 40% of reduction in the V947E protein expression level (Figures 3A,B). In addition, biotinylation experiments revealed an altered V947E protein distribution between the plasma membrane and cytoplasmic compartments. The fraction of the V947E mutant channel reaching the plasma membrane was reduced by about 40% compared to WT, in full agreement with the reduction of V947E chloride currents. Incubation of V947E-transfected cells with 50 µM NFA for 24 h induced an increase in V947E total protein expression, restoring the mutant protein amount to a level similar to those of WT (Figure 3A,B). In parallel, an enhanced V947E protein expression at the plasma membrane level was detected in response to 50 µM NFA incubation, showing a subcellular distribution more similar to that of WT (Figure 3C,D).

In addition, we tested the chaperone activity of 9-anthracene carboxylic acid (9-AC) on WT and A531V-transfected cells. This drug inhibited human ClC-1 channels with an IC_50_ of 24 ± 5 μM at −90 mV (Altamura et al., 2018b). After 24 h incubation with 30 μM 9-AC, the current density of A531V channels was increased by about 60%, whereas no significant difference was observed for WT (Supplementary Figure S3). The voltage dependence did not alter, suggesting that treatment with 9-AC increased protein membrane expression without altering the function of ClC-1 (Supplementary Figure S3).

## 4 Discussion

The pathomechanism of a number of MC mutations has been attributed to disruption of the proteostasis of the ClC-1 chloride channel, which entails a reduction of ClC-1 protein abundance at the plasma membrane.

Like other membrane proteins, the mechanisms governing ClC-1 proteostasis comprise translational and post-translational regulation, which control the concentration, conformation, interaction, and subcellular localization of ClC-1 proteins. These proteins are exposed to different post-translational controls, which include protein quality control at the endoplasmic reticulum (ER quality control), ClC-1 membrane trafficking, and protein turnover at the plasma membrane.

As far as these processes are concerned, the most comprehensive analyses regarded *p*. A531V, a recessively inherited mutation found in northern Finland, Scandinavia, and more recently in Italy (Papponen et al., 1999; Sun et al., 2001; Brugnoni et al., 2013). This ClC-1 mutant displayed a dramatically diminished whole-cell chloride current density, a striking reduction in the total protein expression level, and a significantly shorter protein half-life (Desaphy et al., 2013; Lee et al., 2013). It was thus suggested that the A531V mutation severely impairs protein folding, which renders most of the mutant proteins undesirable for the quality control systems at the ER, Golgi, and plasma membrane, shifting ClC-1 proteostasis toward the degradation pathway (Chen et al., 2015; Peng et al., 2016). Recently, the functional characterization of MC mutations located in the C-terminus of the channels has allowed us to identify new ClC-1 mutations with a likely defect in proteostasis. The valine to glutamate substitution at residue 947 resulted in a substantial reduction of chloride current density, despite a voltage dependence of channel activation indistinguishable from that of WT (Altamura et al., 2018a). In this study, the combination of functional studies with biochemical experiments revealed a decrease in total V947E protein levels and an impaired distribution of the ClC-1 channel between the cytoplasm and plasma membrane, confirming a deficiency in proteostasis.

A promising strategy to treat protein-misfolding channelopathies consists in the use of pharmacological chaperones. These molecules are reversible small compounds able to bind misfolded proteins, allowing the escape from the ER quality control and routing of the target protein towards the plasma membrane (Tran et al., 2020). Several pharmacological chaperones have entered the clinical practice in rare diseases caused primarily by protein instability. For instance, VX-809 (lumacaftor) and the newest VX-661 (tezacaftor) have been approved by FDA as pharmacological chaperones for the treatment of cystic fibrosis, acting on the F508del mutation of CFTR associated with protein misfolding (Southern et al., 2020).

Regarding ClC-1, the availability of compound selectively targeting ClC-1 proteins is currently limited. Only ClC-1 blockers have been discovered, such as 9AC and NFA, whereas no direct activator is known (Estévez et al., 2003; Altamura et al., 2018b). NFA is a small molecule belonging to the nonsteroidal anti-inflammatory drugs, commonly used clinically for the treatment of rheumatoid arthritis and the relief of chronic and acute pain conditions (Shichikawa et al., 1999). We previously showed that NFA inhibits the ClC-1 channel quickly and reversibly, probably by binding to a preferential pocket containing the two amino acidic residues R421 and F484 (Altamura et al., 2018b). Most probably, 9-AC binds to the same caver as NFA (Estévez et al., 2003; Altamura et al., 2018b; Wang et al., 2019). In the ClC-1 homology model, A531 and V947 residues are positioned far from the binding pocket of drugs, suggesting that these mutations may not alter binding to the protein. Accordingly, NFA reversibly inhibited A531V and V947E chloride currents to a similar extent as WT. The incubation of transfected cells with NFA for 24 h determined an increase in A531V and V947E current density, resulting from the rescue of ClC-1 protein expression and subcellular distribution. A similar effect was obtained with 9-AC on A531V currents. Because of the pleiotropic effects of NFA on various ion channels and intracellular signaling (Scott-Ward et al., 2004; Balderas et al., 2012; Marwaha et al., 2016), we cannot exclude that part of the chaperone activity of NFA on trafficking-defective ClC-1 channels may be indirect. However, since both NFA and 9-AC had chaperone activities, it is likely that binding to ClC-1 channels is involved in the chaperone activity.

Thus, ClC-1 channel ligands can work as pharmacological chaperones to rescue pathogenic trafficking-defective ClC-1 variants.

Despite the use of transfected HEK cell lines that could present some limitations, different studies have previously elucidated the proteostasis of ClC-1 channels in HEK cells and demonstrated the existence of a similar network in the rat skeletal muscle (Peng et al., 2016, 2018; Jeng et al., 2020). Nevertheless, further studies on primary human muscle cells or iPSC-derived muscle cells from patients carrying such mutations would be of great interest to support the hypothesis of the chaperone activity of NFA.

Although 9-AC lacks direct therapeutic interest because of its toxicity, NFA is a widely used drug, available over the counter in many countries. Importantly, the concentration of NFA exerting the chaperone effect (50 µM) falls within the range of therapeutic blood concentrations in humans (2–35 μg/ml corresponding to 7–124 µM) (Schulz et al., 2012), suggesting a possible repurposing. However, NFA acutely blocks ClC-1 chloride currents; thus, the net effect of the drug on skeletal muscle conductance *in vivo* remains to be investigated. To this issue, we may postulate that longer exposure at lower doses or small chemical modifications of NFA might prevent its blocking activity but preserve its chaperone effect allowing protein binding, stabilization, and trafficking. Several experiments have been carried out following this direction: for instance, in long QT syndrome, the use of terfenadine carboxylate (fexofenadine), which differs from terfenadine by only a single carboxylic group, induced the recovery of trafficking defective HERG mutants with lower potency for the HERG block (Rajamani et al., 2002). Furthermore, the recent clarification of the binding sites of 9-AC and NFA in ClC-1 will pave the way for the rational design and screening of novel ClC-specific compounds useful for the treatment of ClC-1-associated diseases (Altamura et al., 2018b).

It is worth noting that a number of trafficking-defective ClC-1 channels might remain functionally inactive once rescued to the plasma membrane. For instance, the G411C mutation disrupted the folding of the ClC-1 protein, making the chloride channels both trafficking-defective and nonfunctional (Altamura et al., 2020b). In this case, the drug should be able to not only restore cell surface expression but also improve channel functions. Hence, it is important to increase the comprehension of the molecular mechanisms underlying MC mutations to address the discovery of drugs targeting specific mutant channel defects.

In conclusion, this study provides a proof of concept for the use of pharmacological chaperones in myotonic patients carrying trafficking-defective ClC-1 mutations. The next step will be to verify whether such an effect may be replicated in primary human muscle cells or iPSC-derived muscle cells from trafficking-defective ClC-1 patients as well as *in vivo*.

## Data Availability

The raw data supporting the conclusions of this manuscript will be made available by the authors, without undue reservation.

## References

[B1] Altamura C. Desaphy J. F. Conte D. De Luca A. Imbrici P. (2020a). Skeletal muscle ClC-1 chloride channels in health and diseases. Pflugers Arch. 472 (7), 961–975. 10.1007/s00424-020-02376-3 32361781

[B2] Altamura C. Ivanova E. A. Imbrici P. Conte E. Camerino G. M. Dadali E. L. (2020b). Pathomechanisms of a CLCN1 mutation found in a Russian family suffering from becker's myotonia. Front. Neurol. 11, 1019. 10.3389/fneur.2020.01019 33013670 PMC7500137

[B3] Altamura C. Lucchiari S. Sahbani D. Ulzi G. Comi G. P. D’Ambrosio P. (2018a). The analysis of myotonia congenita mutations discloses functional clusters of amino acids within the CBS2 domain and the C-terminal peptide of the ClC-1 channel. Hum. Mutat. 39, 1273–1283. 10.1002/humu.23581 29935101

[B37] Altamura C. Mangiatordi G. F. Nicolotti O. Sahbani D. Farinato A. Leonetti F. (2018b). Mapping ligand binding pockets in chloride ClC-1 channels through an integrated in silico and experimental approach using anthracene-9-carboxylic acid and niflumic acid. Br. J. Pharmacol. 175 (10), 1770–1780. 10.1111/bph.14192 29500929 PMC5913395

[B4] Andersen G. Hedermann G. Witting N. Duno M. Andersen H. Vissing J. (2017). The antimyotonic effect of lamotrigine in non-dystrophic myotonias: A double-blind randomized study. Brain 140 (9), 2295–2305. 10.1093/brain/awx192 29050397

[B5] Arnold W. D. Kline D. Sanderson A. Hawash A. A. Bartlett A. Novak K. R. (2017). Open-label trial of ranolazine for the treatment of myotonia congenita. Neurology 89 (7), 710–713. 10.1212/WNL.0000000000004229 28710329 PMC5562961

[B6] Balderas E. Ateaga-Tlecuitl R. Rivera M. Gomora J. C. Darszon A. (2012). Niflumic acid blocks native and recombinant T-type channels. J. Cell. Physiol. 227 (6), 2542–2555. 10.1002/jcp.22992 21898399 PMC4146346

[B7] Brugnoni R. Kapetis D. Imbrici P. Pessia M. Canioni E. Colleoni L. (2013). A large cohort of myotonia congenita probands: Novel mutations and a high-frequency mutation region in exons 4 and 5 of the CLCN1 gene. J. Hum. Genet. 58 (9), 581–587. 10.1038/jhg.2013.58 23739125

[B8] Chen Y. A. Peng Y. J. Hu M. C. Huang J. J. Chien Y. C. Wu J. T. (2015). The Cullin 4A/B-DDB1-Cereblon E3 ubiquitin ligase complex mediates the degradation of CLC-1 chloride channels. Sci. Rep. 5, 10667. 10.1038/srep10667 26021757 PMC4448132

[B9] Desaphy J. F. Altamura C. Vicart S. Fontaine B. (2021). Targeted therapies for skeletal muscle ion channelopathies: Systematic review and steps towards precision medicine. J. Neuromuscul. Dis. 8 (3), 357–381. 10.3233/JND-200582 33325393 PMC8203248

[B10] Desaphy J. F. Gramegna G. Altamura C. Dinardo M. M. Imbrici P. George A. L. Jr. (2013). Functional characterization of ClC-1 mutations from patients affected by recessive myotonia congenita presenting with different clinical phenotypes. Exp. Neurol. 248, 530–540. 10.1016/j.expneurol.2013.07.018 23933576 PMC3781327

[B11] Dunø M. Vissing J. (2005). “Myotonia congenita,” in Gene Reviews. Editors Adam M. P. Mirzaa G. M. Pagon R. A. Wallace S. E. Bean L. J. H. Gripp K. W.. (Seattle, WA: University of Washington), 1993–2022. 20301529

[B12] Estévez R. Schroeder B. C. Accardi A. Jentsch T. J. Pusch M. (2003). Conservation of chloride channel structure revealed by an inhibitor binding site in ClC-1. Neuron 38, 47–59. 10.1016/s0896-6273(03)00168-5 12691663

[B13] Human Gene Mutation Database (2022). *CLCN1* gene. AvaliableAt: http://www.hgmd.cf.ac.uk/ac/index.php (Accessed May 16, 2022).

[B14] Imbrici P. Altamura C. Camerino G. M. Mangiatordi G. F. Conte E. Maggi L. (2016). Multidisciplinary study of a new ClC-1 mutation causing myotonia congenita: A paradigm to understand and treat ion channelopathies. FASEB J. 30 (10), 3285–3295. 10.1096/fj.201500079R 27324117 PMC5024700

[B15] Imbrici P. Maggi L. Mangiatordi G. F. Dinardo M. M. Altamura C. Brugnoni R. (2015). ClC-1 mutations in myotonia congenita patients: Insights into molecular gating mechanisms and genotype-phenotype correlation. J. Physiol. 593, 4181–4199. 10.1113/JP270358 26096614 PMC4594292

[B16] Jeng C. J. Fu S. J. You C. Y. Peng Y. J. Hsiao C. T. Chen T. Y. (2020). Defective gating and proteostasis of human ClC-1 chloride channel: Molecular pathophysiology of myotonia congenita. Front. Neurol. 11, 76. 10.3389/fneur.2020.00076 32117034 PMC7026490

[B17] Lee T. T. Zhang X. D. Chuang C. C. Chen J. J. Chen Y. A. Chen S. C. (2013). Myotonia congenita mutation enhances the degradation of human CLC-1 chloride channels. PLoS One 8 (2), e55930. 10.1371/journal.pone.0055930 23424641 PMC3570542

[B18] Lo Monaco M. D’Amico A. Luigetti M. Desaphy J. F. Modoni A. (2015). Effect of mexiletine on transitory depression of compound motor action potential in recessive myotonia congenita. Clin. Neurophysiol. 126 (2), 399–403. 10.1016/j.clinph.2014.06.008 25065301

[B19] Maggi L. Bonanno S. Altamura C. Desaphy J. F. (2021). ion channel gene mutations causing skeletal muscle disorders: Pathomechanisms and opportunities for therapy. Cells 10 (6), 1521. 10.3390/cells10061521 34208776 PMC8234207

[B20] Marwaha L. Bansal Y. Singh R. Saroj P. Sodhi R. K. Kuhad A. (2016). Niflumic acid, a TRPV1 channel modulator, ameliorates stavudine-induced neuropathic pain. Inflammopharmacology 24 (6), 319–334. 10.1007/s10787-016-0285-0 27757590

[B21] Papponen H. Nissinen M. Kaisto T. Myllyla V. V. Myllyla R. Metsikko K. (2008). F413C and A531V but not R894X myotonia congenita mutations cause defective endoplasmic reticulum export of the muscle-specific chloride channel CLC-1. Muscle Nerve 37, 317–325. 10.1002/mus.20922 17990293

[B22] Papponen H. Toppinen T. Baumann P. Myllyla V. Leisti J. Kuivaniemi H. (1999). Founder mutations and the high prevalence of myotonia congenita in northern Finland. Neurology 53, 297–302. 10.1212/WNL.53.2.297 10430417

[B23] Peng Y. J. Huang J. J. Wu H. H. Hsieh H. Y. Wu C. Y. Chen S. C. (2016). Regulation of CLC-1 chloride channel biosynthesis by FKBP8 and Hsp90β. Sci. Rep. 6, 32444. 10.1038/srep32444 27580824 PMC5007535

[B24] Peng Y. J. Lee Y. C. Fu S. J. Chien Y. C. Liao Y. F. Chen T. Y. (2018). FKBP8 enhances protein stability of the CLC-1 chloride channel at the plasma membrane. Int. J. Mol. Sci. 19 (12), 3783. 10.3390/ijms19123783 PMC6320802 30487393

[B25] Rajamani S. Anderson C. L. Anson B. D. January C. T. (2002). Pharmacological rescue of human K(+) channel long-QT2 mutations: Human ether-a-go-go-related gene rescue without block. Circulation 105 (24), 2830–2835. 10.1161/01.cir.0000019513.50928.74 12070109

[B26] Schulz M. Iwersen-Bergmann S. Andresen H. Schmoldt A. (2012). Therapeutic and toxic blood concentrations of nearly 1,000 drugs and other xenobiotics. Crit. Care 16 (4), R136. 10.1186/cc11441 22835221 PMC3580721

[B27] Scott-Ward T. S. Li H. Schmidt A. Cai Z. Sheppard D. N. (2004). Direct block of the cystic fibrosis transmembrane conductance regulator Cl (-) channel by niflumic acid. Mol. Membr. Biol. 21 (1), 27–38. 10.1080/09687680310001597758 14668136

[B28] Shichikawa K. Inoue K. Hirota S. Maeda A. Ota H. Kimura M. (1999). Changes in the incidence and prevalence of rheumatoid arthritis in Kamitonda, Wakayama, Japan, 1965-1996. Ann. Rheum. Dis. 58 (12), 751–756. 10.1136/ard.58.12.751 10577961 PMC1752814

[B29] Southern K. W. Murphy J. Sinha I. P. Nevitt S. J. (2020). Corrector therapies (with or without potentiators) for people with cystic fibrosis with class II CFTR gene variants (most commonly F508del). Cochrane Database Syst. Rev. 12 (12), CD010966. 10.1002/14651858.CD010966.pub3 33331662 PMC8094390

[B30] Statland J. M. Bundy B. N. Wang Y. Rayan D. R. Trivedi J. R. Sansone V. A. (2012). Mexiletine for symptoms and signs of myotonia in nondystrophic myotonia: A randomized controlled trial. JAMA 308 (13), 1357–1365. 10.1001/jama.2012.12607 23032552 PMC3564227

[B31] Stunnenberg B. C. LoRusso S. Arnold W. D. Barohn R. J. Cannon S. C. Fontaine B. (2020). Guidelines on clinical presentation and management of nondystrophic myotonias. Muscle Nerve 62 (4), 430–444. 10.1002/mus.26887 32270509 PMC8117169

[B32] Stunnenberg B. C. Raaphorst J. Groenewoud H. M. Statland J. M. Griggs R. C. Woertman W. (2018). Effect of mexiletine on muscle stiffness in patients with nondystrophic myotonia evaluated using aggregated N-of-1 trials. JAMA 320 (22), 2344–2353. 10.1001/jama.2018.18020 30535218 PMC6583079

[B33] Sun C. Tranebjaerg L. Torbergsen T. Holmgren G. Van Ghelue M. (2001). Spectrumof CLCN1 mutations in patients with myotonia congenita in Northern Scandinavia. Eur. J. Hum. Genet. 9, 903–909. 10.1038/sj.ejhg.5200736 11840191

[B34] Tran M. L. Génisson Y. Ballereau S. Dehoux C. (2020). Second-generation pharmacological chaperones: Beyond inhibitors. Molecules 25 (14), 3145. 10.3390/molecules25143145 PMC7397201 32660097

[B35] Wang K. Preisler S. S. Zhang L. Cui Y. Missel J. W. Grønberg C. (2019). Structure of the human ClC-1 chloride channel. PLoS Biol. 17 (4), e3000218. 10.1371/journal.pbio.3000218 31022181 PMC6483157

[B36] Weinberger S. Wojciechowski D. Sternberg D. Lehmann-Horn F. Jurkat-Rott K. Becher T. (2012). Disease-causing mutations C277R and C277Y modify gating of human ClC-1 chloride channels in myotonia congenita. J. Physiol. 590 (15), 3449–3464. 10.1113/jphysiol.2012.232785 22641783 PMC3547262

