# Supplementary material for: Chaperone activity of niflumic acid on ClC-1 chloride channel mutants causing myotonia congenita
Source: Front Pharmacol. 2022 Aug 11;13:958196. doi: 10.3389/fphar.2022.958196 (PMC9403836; doi:10.3389/fphar.2022.958196)
Supplement: Supplementary file 1 [file DataSheet1.PDF]

Supplementary Material

Supplementary Figures

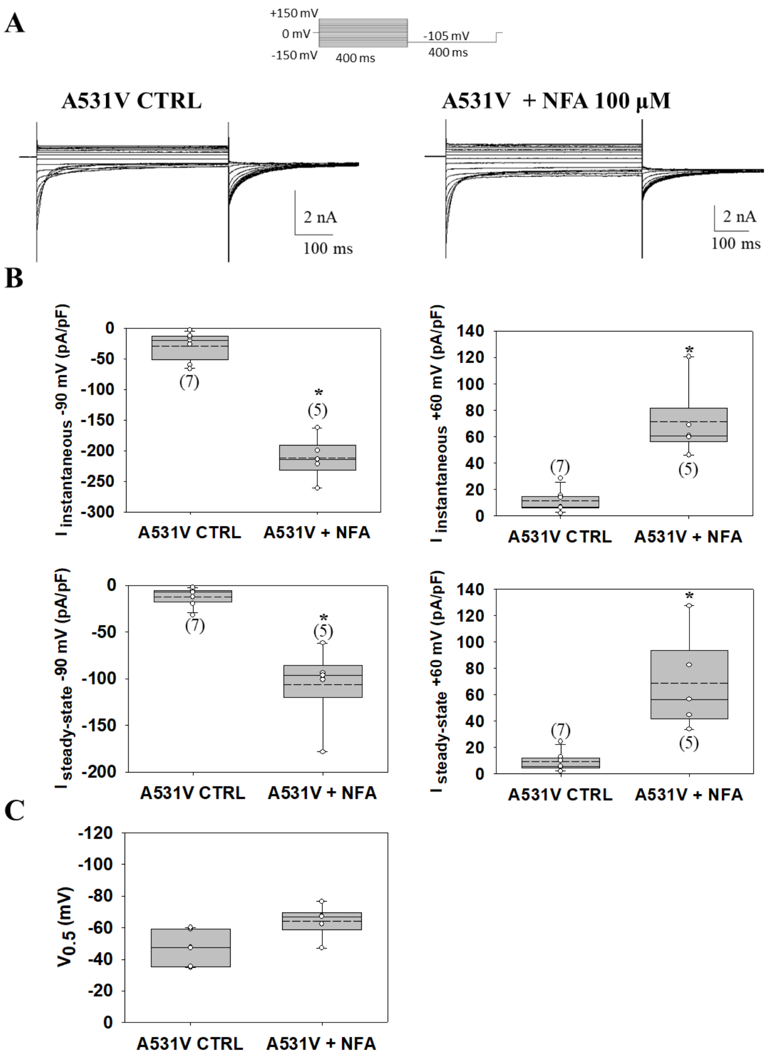

Supplementary Figure 1. Chaperone effect of 100 µM NFA on A531V ClC-1 chloride channels. **A.** Representative traces of A531V chloride currents before and after the incubation of 100 µM NFA for 24h. **B.** Values of instantaneous and steady-state chloride current of A531V mutant measured at -90 and +60 mV before and after the incubation with NFA 100 µM. **C.** Values of $V_{0.5}$ (half-maximal activation potential) of A531V channel before and after the incubation of NFA. Each dot represented $V_{0.5}$ value obtaining from single recording. ~~For each recording, the distribution of P open was plotted and the $V_{0.5}$ values reported correspond to the resulting mean values ± SEM.~~
Data were shown as box-and-whisker plots. Median (solid line), mean (dash line) and 10th, 25th, 75th, and 90th percentiles were indicated. Number of examined cells were reported in brackets.

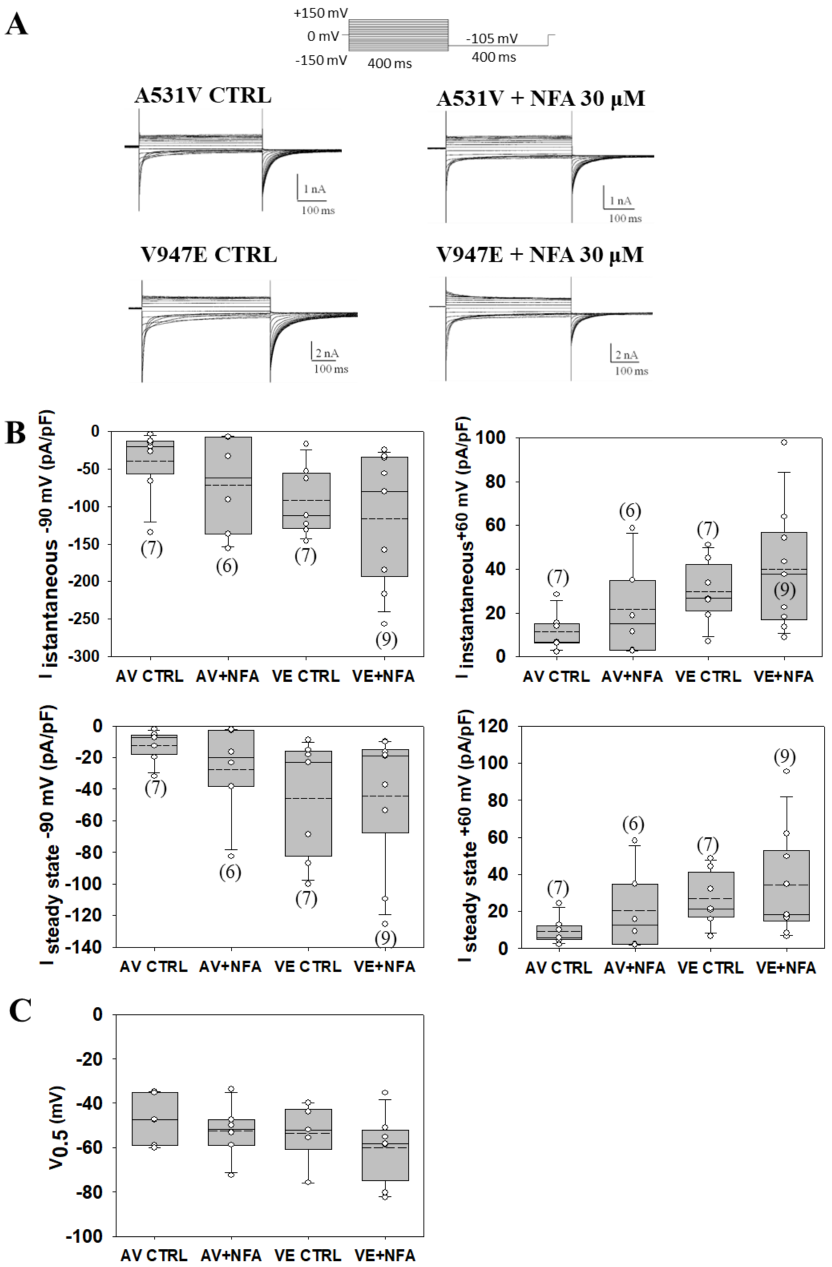

Supplementary Figure 2. Chaperone effect of 30 µM NFA on A531V and V947E ClC-1 chloride channels. **A.** Representative traces of A531V and V947E chloride currents before and after the incubation of 30 µM NFA for 24h. **B.** Values of instantaneous and steady-state chloride current of A531V and V947E mutants measured at -90 and +60 mV before and after the incubation with NFA 30 µM. **C.** Values of $V_{0.5}$ (half-maximal activation potential) of A531V and V947E channels before and after the incubation of NFA. Each dot represented $V_{0.5}$ value obtaining from single recording. ~~For each recording, the distribution of P open was plotted and the $V_{0.5}$ values reported correspond to the resulting mean values ± SEM.~~
Data were shown as box-and-whisker plots. Median (solid line), mean (dash line) and 10th, 25th, 75th, and 90th percentiles were indicated. Number of examined cells were reported in brackets.

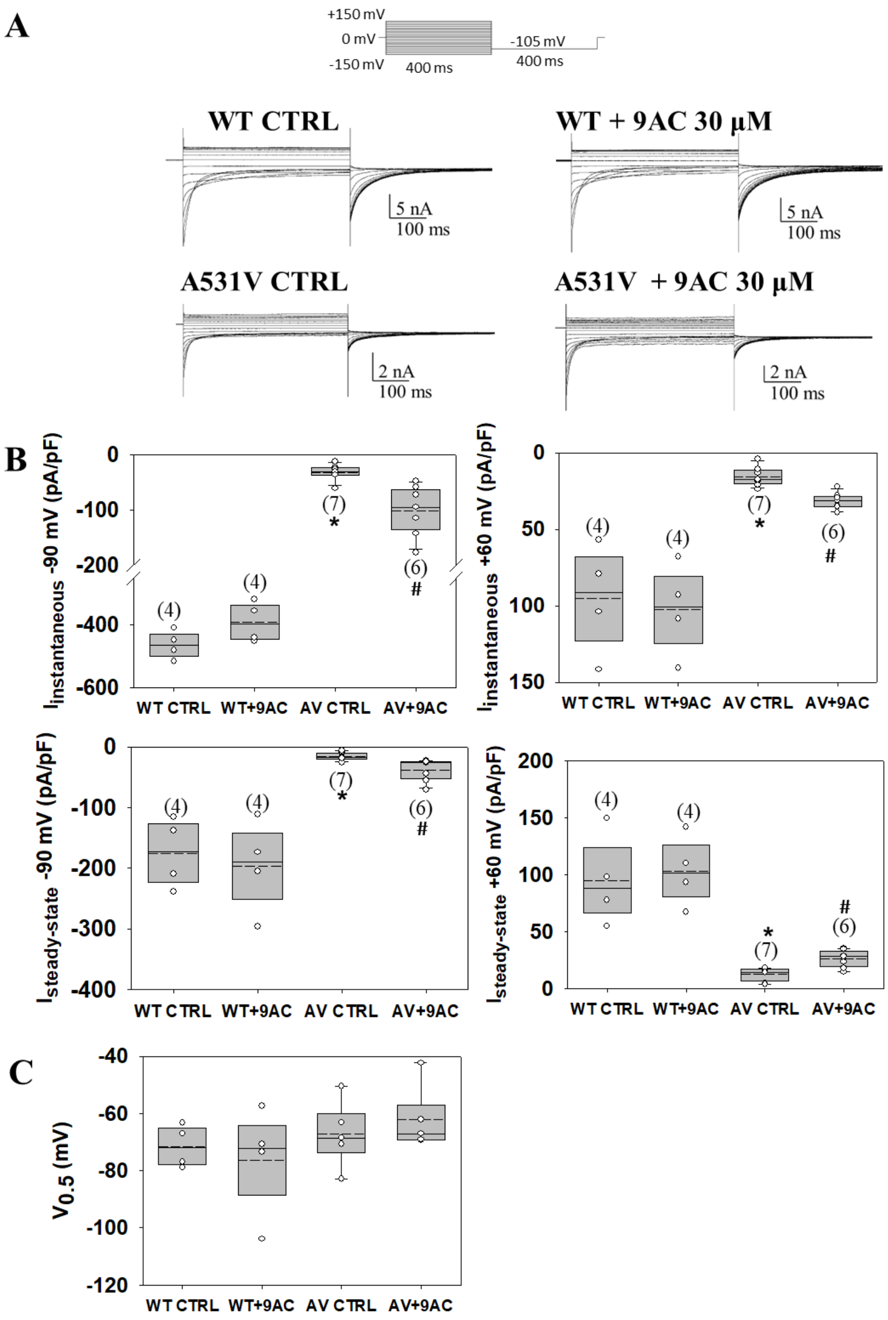

Supplementary Figure 3. Chaperone effect of 30 µM 9AC on WT and A531V ClC-1 chloride channels.

A. Representative traces of WT and A531V chloride currents before and after the incubation of 30 µM 9AC for 24h. **B.** Values of steady-state chloride current of WT and A531V channel measured at -90 and +60 mV before and after the incubation with 9AC 30 µM. **C.** Mean values of $V_{0.5}$ (half-maximal activation potential) of WT and A531V channels before and after the incubation of 9AC. Each dot represented $V_{0.5}$ value obtaining from single recording. ~~For each recording, the distribution of P open was plotted and the $V_{0.5}$ values reported correspond to the resulting mean values ± SEM.~~

Data were shown as box-and-whisker plots. Median (solid line), mean (dash line) and 10th, 25th, 75th, and 90th percentiles were indicated. Number of examined cells were reported in brackets.

(*at least $p< 0.05$ vs WT CTRL, # at least $p< 0.05$ vs AV CTRL).
